# Deciphering the Combined Effects of Hydroxysafflor Yellow A and Calycosin Through Coupled PK–PD Modeling

**DOI:** 10.3390/biology15171543

**Published:** 2026-09-04

**Authors:** Yanxuan Hu, Xixi Zhao, Weifeng Jin, Li Yu

**Affiliations:** 1 The Second Clinical Medical College, Zhejiang Chinese Medical University, Hangzhou 310053, China; 2 Laboratory Animal Research Center, Academy of Chinese Medical Sciences, Zhejiang Chinese Medical University, Hangzhou 310053, China; 3 School of Pharmaceutical Sciences, Zhejiang Chinese Medical University, Hangzhou 310053, China; 4 School of Basic Medical Sciences, Zhejiang Chinese Medical University, Hangzhou 310053, China

**Keywords:** coupled PK model, coupled PK-PD model, factorial design, hierarchical optimization, component interaction

## Abstract

To quantitatively characterize the pharmacokinetic and pharmacodynamic interactions between Hydroxysafflor Yellow A (HSYA) and Calycosin (CA) in cerebral ischemia–reperfusion injury (CIRI), a factorial experimental design was combined with a hierarchical coupled PK-PD modeling framework. The coupled PK model adequately described the concentration-time profiles of HSYA and CA under co-administration, with *R*^2^ values of 0.83 and 0.86, respectively, while the PK-PD model showed good fitting performance for Caspase-3 and HIF-1α, with all *R*^2^ values exceeding 0.97. Structural identifiability analysis showed that the newly introduced PK and PD coupling parameters were globally identifiable, and their Bootstrap 95% confidence intervals excluded zero. After Holm-Bonferroni correction, *AUC*_0-*t*_, *AUC*_0-*∞*_ and *CL* differed significantly between single and combined administration, whereas MRT did not. The model-derived relative contribution weights of HSYA and CA were quantified for both Caspase-3 and HIF-1α, with uncertainty assessed using Bootstrap 95% confidence intervals. Overall, the proposed coupled PK-PD framework provides a quantitative approach for characterizing component interactions and relative pharmacodynamic contributions in multi-component systems.

## Simple Summary

Safflower and astragalus are two medicinal herbs often given together in traditional Chinese medicine to treat brain injury, and two of their active ingredients are hydroxysafflor yellow A and calycosin. Using rats with brain damage caused by a temporary interruption of blood flow, this study asked whether and how these two compounds affect each other’s behavior in the body and how much each contributes to the combined treatment effect on two markers of brain-cell injury. We built a computer-based model that tracks how the two compounds are handled by the body and how they act together on these markers. The model reproduced the observed drug levels and marker responses well and showed that the two compounds influence each other’s removal from the body, while their separate contributions to the overall effect could be quantified. Such a modeling approach may help in designing and optimizing multi-component drug combinations.

## 1. Introduction

Pharmacokinetic-pharmacodynamic (PK-PD) modeling quantitatively describes the relationship between plasma drug concentrations and their pharmacological effects. To account for delays between drug concentrations in plasma and their effect-site concentrations, the effect compartment model was designed, enabling more accurate linkage between PK and PD profiles [1]. In recent years, more and more studies have attempted to apply PK-PD modeling to the field of traditional Chinese medicine (TCM) to reveal the multi-component and multi-target pharmacodynamic mechanisms. However, the existing models still have obvious deficiencies in capturing the synergistic effects of multi-components, which limits the in-depth understanding of the pharmacodynamic mechanism of TCM and the ability to scientifically evaluate the efficacy of drug combinations.

Such complexity is mainly reflected in several aspects. The absorption, distribution, metabolism, and excretion of individual components may be influenced differently by the presence of other components [2,3]. A time delay may also occur between drug concentration and pharmacodynamic response [4]. In addition, synergistic or antagonistic interactions may arise among multiple components [5,6]. These challenges highlight the urgent need to adapt and improve traditional PK-PD models so that they can better capture the dynamic changes, multi-target effects, and interactive features of multi-component systems.

More advanced approaches, including mechanism-based PK-PD, PBPK, systems pharmacology/network-based models, and nonlinear mixed-effects models, can address physiological mechanisms, pathway interactions, or population variability, but generally require substantially richer physiological, molecular, tissue-level, or population data. The present framework is therefore intended as an intermediate-complexity complementary approach for explicitly describing component-dependent PK and PD changes under the current experimental setting.

Calycosin (CA) and Hydroxysafflor Yellow A (HSYA), the principal bioactive components of Astragalus and Safflower, have been shown to exhibit therapeutic potential for encephalopathy and related neurological disorders. Their therapeutic effects are mediated through a range of signaling pathways, including JAK2/STAT3 [7], SIRT1 [8], and TLR4 [9]. Our earlier work has demonstrated that co-administration of HSYA and CA significantly influences the PK-PD behaviors in a rat model of cerebral ischemia–reperfusion injury (CIRI) [10]. However, due to the lack of a systematically designed single-component control group, it has not been possible to definitively assess whether there is synergy between the two or to quantify the specific contribution of each to pharmacodynamic indices.

To address these limitations, the effects of HSYA and CA, both individually and in combination, are systematically compared using a factorial design experiment in this study. Specifically, the drug concentrations and pharmacodynamic indicators over time in plasma samples from CIRI rats were measured. To account for the complex PK-PD relationships, a coupled PK-PD modeling framework was presented along with its hierarchical optimization algorithm. By integrating experimental data with novel modeling strategies, the biological relevance and computational accuracy were not only validated, but the mechanistic understanding of how HSYA and CA interact to exert therapeutic effects in CIRI was also deepened. Accordingly, a complementary coupled PK-PD framework for quantitatively investigating multi-component systems is introduced.

## 2. Materials and Methods

### 2.1. Materials and Reagents

Calycosin (CA; purity ≥ 98%, batch number DSTDM001201) was obtained from Chengdu Lemeitian Pharmaceutical Technology Co., Ltd. (Chengdu, China). Hydroxysafflor Yellow A (HSYA; purity ≥ 98%, batch number R19O11F127828) was purchased from Shanghai Yuanye Biotechnology Co., Ltd. (Shanghai, China). Rutin (purity ≥ 98%, batch number 20141027), which served as the internal standard (IS), was provided by Shanghai Jinsui Biotechnology Co., Ltd. (Shanghai, China). ELISA kits for Caspase-3 (batch number 20241210) and HIF-1α (batch number 20241210) were supplied by Jiangsu Mei Biao Biological Technology Co., Ltd. (Yancheng, China).

### 2.2. Experimental Animals

Male Sprague-Dawley (SD) rats weighing between 280 and 310 g were supplied by the Experimental Animal Center of Zhejiang Chinese Medical University (license number: SYXK 2021-0012). Experimental animals were housed under standard conditions and underwent a one-week acclimatization period before the operation. Ethical approval was obtained from the university’s Institutional Animal Care and Use Committee (IACUC-20220606-05). All animal experiments were performed in accordance with the ARRIVE guidelines (Animal Research: Reporting of In Vivo Experiments). Euthanasia of experimental animals was performed in accordance with the American Veterinary Medical Association (AVMA) Guidelines for the Euthanasia of Animals (2020). After completing sample collection, euthanasia of experimental rats was performed by decapitation under anesthesia.

### 2.3. Focal Cerebral Ischemia Modeling

Transient focal cerebral ischemia was induced using the middle cerebral artery occlusion (MCAO) model as described by Longa [11]. In brief, the neck skin of the anesthetized rat was incised to expose the right common carotid artery (CCA), external carotid artery (ECA), and internal carotid artery (ICA). Under the conditions of ligating the CCA and ECA and occluding the ICA with a clip, a 0.26 mm round-tipped nylon monofilament was inserted through a “V”-shaped incision in the CCA and advanced via the ICA to occlude the middle cerebral artery. The ischemic period lasted for 1 h, after which reperfusion was achieved.

### 2.4. Group Assignments and Plasma Sample Detection

Rats were randomly assigned to four experimental groups (*n* = 6 per group): (1) MCAO/R model group, (2) MCAO/R + HSYA group (5 mg/kg), (3) MCAO/R + CA group (8 mg/kg), and (4) MCAO/R + HSYA + CA combined group (5 mg/kg + 8 mg/kg). At 24 h after MCAO/R, hydroxysafflor yellow A (HSYA, 5 mg/kg) and calycosin (CA, 8 mg/kg) were administered via tail vein injection using a PEG400: water (3:7, *v*/*v*) formulation. Plasma samples were collected from the jaw vein at 14 predefined time points (0, 2, 5, 10, 15, 20, 30, 45, 60, 75, 90, 120, 180, and 240 min) post-dosing. It is worth noting that the sample size of *n* = 6 indicates that each treatment group consists of 6 independent experimental animals. At each sampling time point, measurements from the six animals were summarized as mean ± SD. The resulting group-mean concentration–time and effect–time profiles were used for subsequent PK and PK–PD model fitting. The individual-animal data were retained for animal-level Bootstrap resampling.

The plasma supernatant obtained after centrifugation at 4500 rpm for 12 min at 4 °C was split into two portions: one for PK analysis to quantify HSYA and CA concentrations using high-performance liquid chromatography (HPLC), and the other for PD analysis to measure Caspase-3 and HIF-1α expression levels using ELISA kits, according to the provided instructions. In the PK study, after protein precipitation with methanol, plasma samples were centrifuged and concentrated to dryness. And the resulting residue was then reconstituted for HPLC analysis. The analysis was performed on an Agilent 1200 Series HPLC system (Agilent Technologies, Santa Clara, CA, USA) equipped with a variable wavelength detector (VWD) set at 260 nm. Chromatographic separation was carried out on an Eclipse XDB-C_18_ column (5 μm, 4.6 mm × 250 mm) (Agilent Technologies, Waldbronn, Germany). And the limits of detection (LLOD) were 0.315 μg/mL for HSYA and 0.0054 μg/mL for CA. Other methodological details, including sample preparation procedures, internal standard selection, and method validation [10], were performed in accordance with our previously published work.

The previously validated method showed good linearity for HSYA over 0.6–13.2 μg/mL (*R*^2^ = 0.9993) and for CA over 0.015–7.68 μg/mL (*R*^2^ = 0.9999). The intra- and inter-day precision values were below 10%, and the extraction recoveries were 84.18–110.42% for HSYA and 99.25–109.86% for CA. Acceptable stability was also demonstrated under short-term storage, repeated freeze–thaw, and frozen-storage conditions. The LLOD values under the present analytical conditions were 0.315 μg/mL for HSYA and 0.0054 μg/mL for CA.

### 2.5. Coupled PK Model

**Figure 1:**
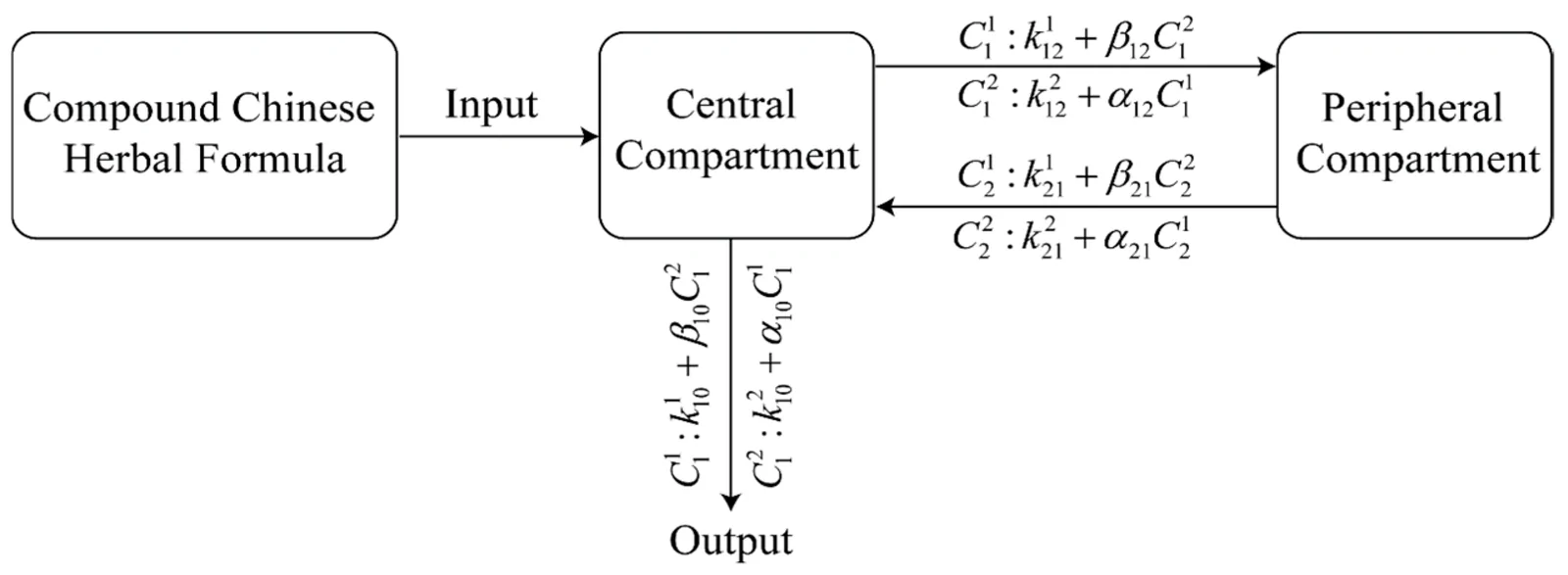
Schematic overview of the coupled PK model.

Conventional two-compartment models generally assume that the pharmacokinetic parameters of each component remain constant and are unaffected by other co-administered components. However, HSYA and CA may influence each other’s distribution and elimination during combined administration. To characterize this interdependence, a coupled pharmacokinetic model was developed while retaining the conventional two-compartment structure for each component, as illustrated in Figure 1.

Within the investigated dose and concentration ranges, the effect of one component on a selected kinetic parameter of the other component was approximated using a linear coupling relationship.

(1) $$\tilde{k_{i}}\left(c_{j}\right)=k_{i}+\beta_{ji}c_{j}$$

where *k_i_* is the baseline kinetic parameter of component *i*, $\tilde{k_{i}}\left(c_{j}\right)$ is its concentration-dependent value under combined administration, *c_j_* denotes the concentration of the interacting component *j*, and *β_ji_* represents the direction and magnitude of the influence of component *j* on component *i*. The complete model formulation and detailed parameter definitions are provided in File S1.

It should be emphasized that the linear coupling term represents a local approximation within the investigated concentration range rather than a general assumption that the interaction remains linear across all exposure levels. This formulation was selected to balance model interpretability, parameter identifiability, and computational feasibility. Nonlinear or saturable interactions may require more flexible coupling functions, particularly at higher concentrations.

Moreover, any continuous and sufficiently smooth nonlinear interaction function can be locally approximated by its first-order Taylor expansion over a restricted concentration range from a mathematical perspective. Accordingly, the estimated coupling coefficient in the present model should be interpreted as the local slope of the concentration-dependent interaction within the observed exposure range rather than as a concentration-independent mechanistic constant.

### 2.6. Coupled PK-PD Model

**Figure 2:**
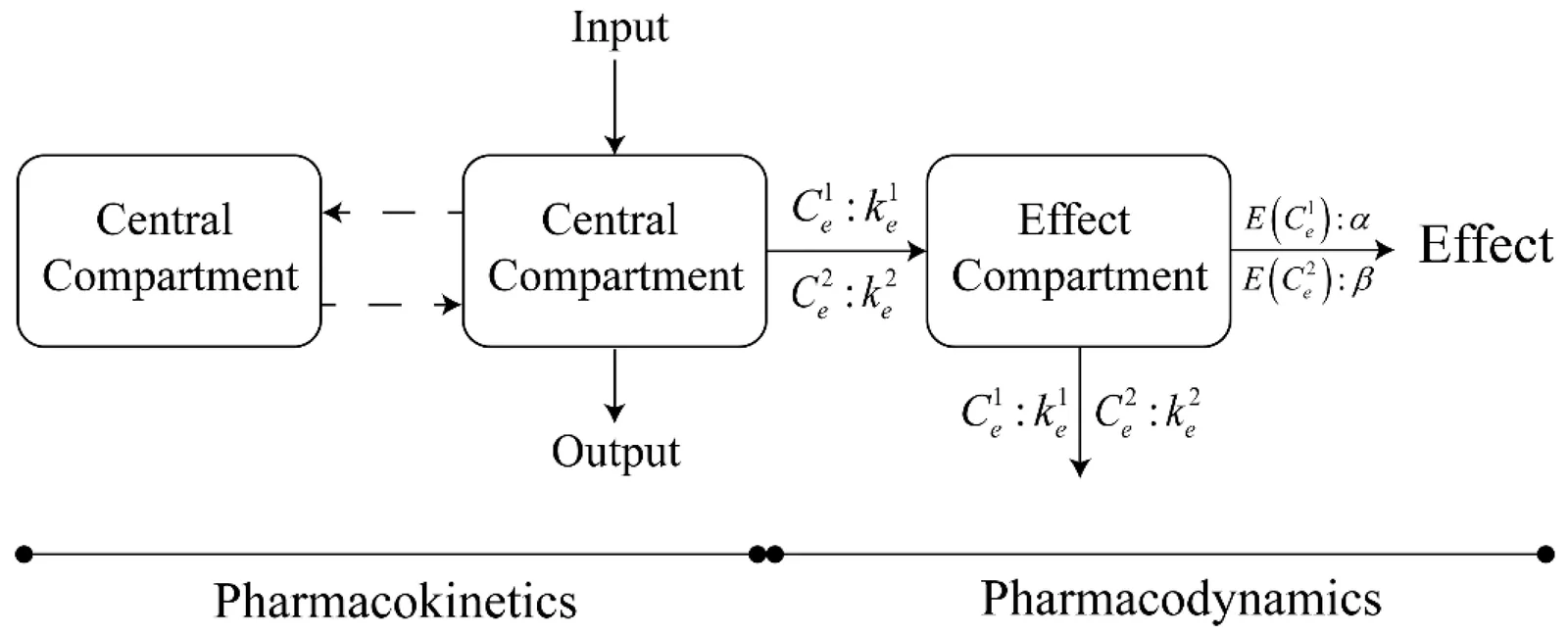
Schematic diagram of the coupled PK-PD model.

Building on the coupled PK framework, a coupled PK–PD model was developed to link the plasma concentration profiles of HSYA and CA with their pharmacodynamic responses. This model integrates two key assumptions: (1) a temporal lag exists between plasma drug concentration and pharmacological effect [1]. (2) the pharmacodynamic contributions of each compound are independently exerted [12,13]. Therefore, the model consists of three interconnected components, including an effect-compartment module, an indirect-response module, and a pharmacodynamic coupling module, as schematically illustrated in Figure 2.

The effect-compartment module was introduced to account for the temporal delay between plasma concentrations and pharmacological responses. For each component, the effect-compartment concentration was linked to the central-compartment concentration through a first-order equilibration process as:

(2) $$\frac{dC_{e}^{i}}{dt}=k_{e}^{i}\left(C_{1}^{i}\;-\;C_{e}^{i}\right).$$

To characterize multiphasic and non-monotonic time–effect profiles, an indirect response model was further employed as:

(3) $$\frac{dE_{i}}{dt}=k_{in}^{i}\times \left(1+\frac{E_{max}^{i}C_{e}^{i}}{EC_{50}^{i}+C_{e}^{i}}\right)\;-\;k_{out}^{i}\times E_{i}.$$

The combined pharmacodynamic response was then expressed as a linear combination.

(4) $$E=\alpha E_{1}+\beta E_{2}.$$

The complete coupled PK–PD model and detailed parameter definitions are illustrated in File S2. For PK-PD model fitting, the AUEC values derived from the pharmacodynamic observations were log-transformed before parameter estimation, and the corresponding model fitting and residual analyses were performed on the log (AUEC) scale.

A central rationale for multi-component formulations in TCM lies in their capacity to enhance therapeutic efficacy through synergistic interactions. The coupling parameters quantify the direction and magnitude of model-level component interactions and should not, by themselves, be interpreted as evidence of pharmacological synergy. To quantitatively assess each component’s contribution within the combined therapeutic effect, a mathematical quantification is provided as:

(5) $$\eta_{A}=\frac{\alpha }{\alpha +\beta }\;\mathrm{and}\;\eta_{B}=\frac{\beta }{\alpha +\beta }.$$

### 2.7. Hierarchical Optimization Approach

The nonlinear feature introduced by coupling terms in the model poses substantial computational challenges. However, the system can be decoupled structurally, allowing for a sequential or hierarchical solution approach that is practical for empirical application. Therefore, the coupled PK-PD model is modularized into three sub-models: (1) single-component PK; (2) dual-component coupled PK; (3) dual-component coupled PD. It follows that the complete PK and PD parameters are not estimated simultaneously during a single optimization process.

Specifically, the PK/PD parameters for each compound administered alone were first estimated using analytical methods [14]. These parameters were then used as initial values for fitting the co-administration data using a particle swarm optimization algorithm [15]. This sequential estimation strategy reduced the number of parameters optimized simultaneously and thereby helped limit the risk of overfitting.

**Figure 3:**
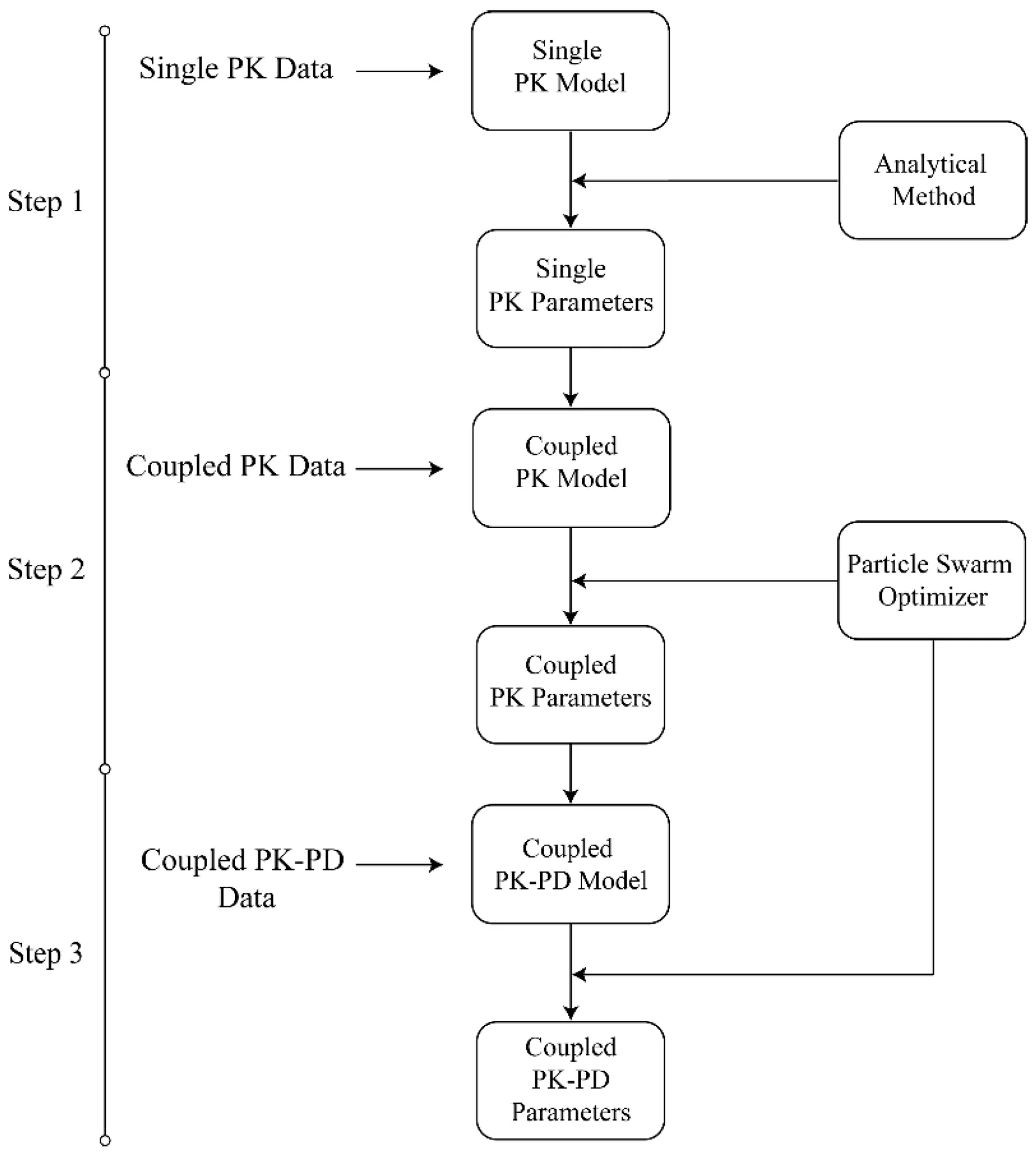
Flowchart of hierarchical optimization approach.

It is worth noting that parameter estimation was based on the longitudinal group-mean concentration–time and effect–time profiles. At each predefined time point, measurements from six animals were summarized to obtain the corresponding mean observation used for model fitting. The overall optimization sequence is summarized in the workflow shown in Figure 3, while the detailed procedure is shown in File S3.

In the process of estimating the parameters of the coupled model, this work introduces physiologically plausible constraints. All PK and PD kinetic parameters with positive definitions are restricted to non-negative values, and the parameter search ranges are set based on the estimation results from the one-component model and the physiological significance of the corresponding parameters. For the PK model, drug transport between the central compartment and the peripheral compartment is constrained by mass balance equations. These constraints ensure that drug concentrations in each compartment remain non-negative during the model calculations and avoid parameter combinations that are inconsistent with pharmacokinetic processes. For the PK-PD model, the effect compartment transport parameters, indirect response parameters, and combined effect parameters were also optimized within preset physiologically plausible ranges. The specific constraints and value ranges for each parameter are provided in File S3.

Furthermore, to assess the uncertainty in the parameter estimates of the coupled PK and PK-PD models, this study employs the Bootstrap resampling method. For each Bootstrap sample, the resampled observations were averaged at each time point to reconstruct a Bootstrap group-mean time profile, and the complete hierarchical model-fitting procedure was then repeated. Parameter estimation is then performed according to the stratified optimization algorithm described above. This process was repeated 100 times to obtain the Bootstrap empirical distributions for each model parameter, with parameter uncertainty expressed as the 95% confidence interval of the Bootstrap distribution. If the 95% Bootstrap confidence interval for a parameter does not include 0, the parameter is considered to have a stable, nonzero effect relative to 0.

### 2.8. Model Structure Identifiability

To evaluate whether the newly introduced parameters in the coupled model can be uniquely determined from the model outputs in theory, this study further conducted a structural identifiability analysis. Given that this study adopted a hierarchical sequential parameter estimation strategy, the structural identifiability analysis was performed separately for the coupled PK model and the coupled PK-PD model, following the actual modeling sequence. At each stage of the analysis, the basic parameters already determined in the previous stage were treated as known quantities, and only the structural identifiability of the newly added coupling parameters at the current stage was examined. Therefore, this study evaluates the conditional structural identifiability of the newly added coupling parameters within the hierarchical estimation framework. To this end, for models described by ordinary differential equations, SIAN was used to perform symbolic structural identifiability analysis [16]. The analysis is based on continuous theoretical model outputs without measurement errors and determines whether the parameters to be estimated are globally structurally identifiable, locally but not globally structurally identifiable, or structurally unidentifiable.

#### 2.8.1. Coupled PK Model

The structural identifiability analysis of the coupled PK model was conducted in accordance with the actual hierarchical parameter estimation process. First, the baseline PK parameters for each component were determined based on data from separate administrations of the two components. During the coupled PK analysis phase, these baseline PK parameters were fixed as known parameters, and only the six concentration-dependent coupling parameters *β*_10_, *β*_12_, *β*_21_, *α*_10_, *α*_12_ and *α*_21_ introduced in the co-administration model were treated as parameters to be analyzed.

The model’s observed outputs are defined as the central compartment concentrations *C*_1_(*t*) and *C*_2_(*t*) of the two components. The state equations, parameter definitions, and specific forms of concentration-dependent rate constants are consistent with those of the coupled PK model. The initial states were determined based on the actual dosing regimen and model settings. The complete state equations, observation equations, known parameters, parameters to be analyzed, and initial conditions were entered into SIAN, with the six coupling parameters simultaneously treated as unknown parameters for structural identifiability analysis.

#### 2.8.2. Coupled PK-PD Model

The coupled PK-PD model employs a hierarchical analysis strategy consistent with the estimation of actual parameters. First, the baseline PD parameters for each of the two components, including the effect-compartment rate constant *k*_e_, the input rate constant *k*_in_, the output rate constant *k*_out_, *E*_max_ and *EC*_50_ for each component and these parameters are held constant during the combined efficacy analysis phase. The concentration-time profiles generated by the coupled PK model serve as inputs to the PD model; therefore, the efficacy profiles *E*_1_(*t*) and *E*(*t*) corresponding to the individual components are treated as known time functions during this analysis phase. For the parameters *α* and *β* in the combined pharmacodynamic model, their structural identifiability is analyzed by examining the functional linear independence of *E*_1_(*t*) and *E*_2_(*t*). That is, by determining whether, given *E*_1_(*t*) and *E*_2_(*t*), there exist different parameter combinations *α* and *β* that can produce exactly the same model output, thereby determining the unique identifiability of *α* and *β*.

### 2.9. Software and Statistical Methods

Data are presented as the mean ± standard deviation (SD). Longitudinal concentration-time and effect-time observations were used for PK and PK-PD model fitting and were not treated as independent observations for between-group comparisons. Pharmacokinetic summary parameters, including AUC, MRT and CL were compared between single and combined administration using two-sided independent-samples tests. For multiple comparisons of PK parameters, *p* values were adjusted using the Holm-Bonferroni method, with an adjusted *p* < 0.05 considered statistically significant.

Model parameter uncertainty was assessed using Bootstrap resampling with animals as the resampling unit, and parameter estimates are reported with 95% Bootstrap confidence intervals. Model fitting was evaluated using *R*^2^ and residual diagnostics. Residual normality was assessed using the Shapiro–Wilk test, deviation of the residual mean from zero using a one-sample t-test, and heteroscedasticity using the Breusch-Pagan test.

All data analysis, numerical computation of models, and optimization were done in the Python 3.12 (Python Software Foundation, Wilmington, DE, USA) environment, mainly using NumPy, Pandas, Openpyxl, SciPy, Pyswarms, Matplotlib, Seaborn and other third-party libraries.

## 3. Results

### 3.1. Experimental Results

HPLC was used to quantify plasma concentrations of HSYA and CA at multiple time points across all groups, and the experimental data are displayed in Table 1.

Additionally, plasma levels of Caspase-3 and HIF-1α were measured using commercial ELISA kits at corresponding time intervals, as shown in Table 2.

### 3.2. Coupled PK Model

Under hierarchical estimation conditions with fixed basic PK parameters, a structural identifiability analysis was conducted on the six newly introduced concentration-dependent coupling parameters in the coupled PK model. The SIAN analysis results show that *β*_10_, *β*_12_, *β*_21_, *α*_10_, *α*_12_, *α*_21_ were all determined to be globally identifiable parameters, and no locally but not globally identifiable parameters or structurally unidentifiable parameters were found. These results indicate that, given known baseline PK parameters, the six coupling parameters can be uniquely determined from the model output; there are no multiple mathematically equivalent parameter combinations that yield the same model output.

**Figure 4:**
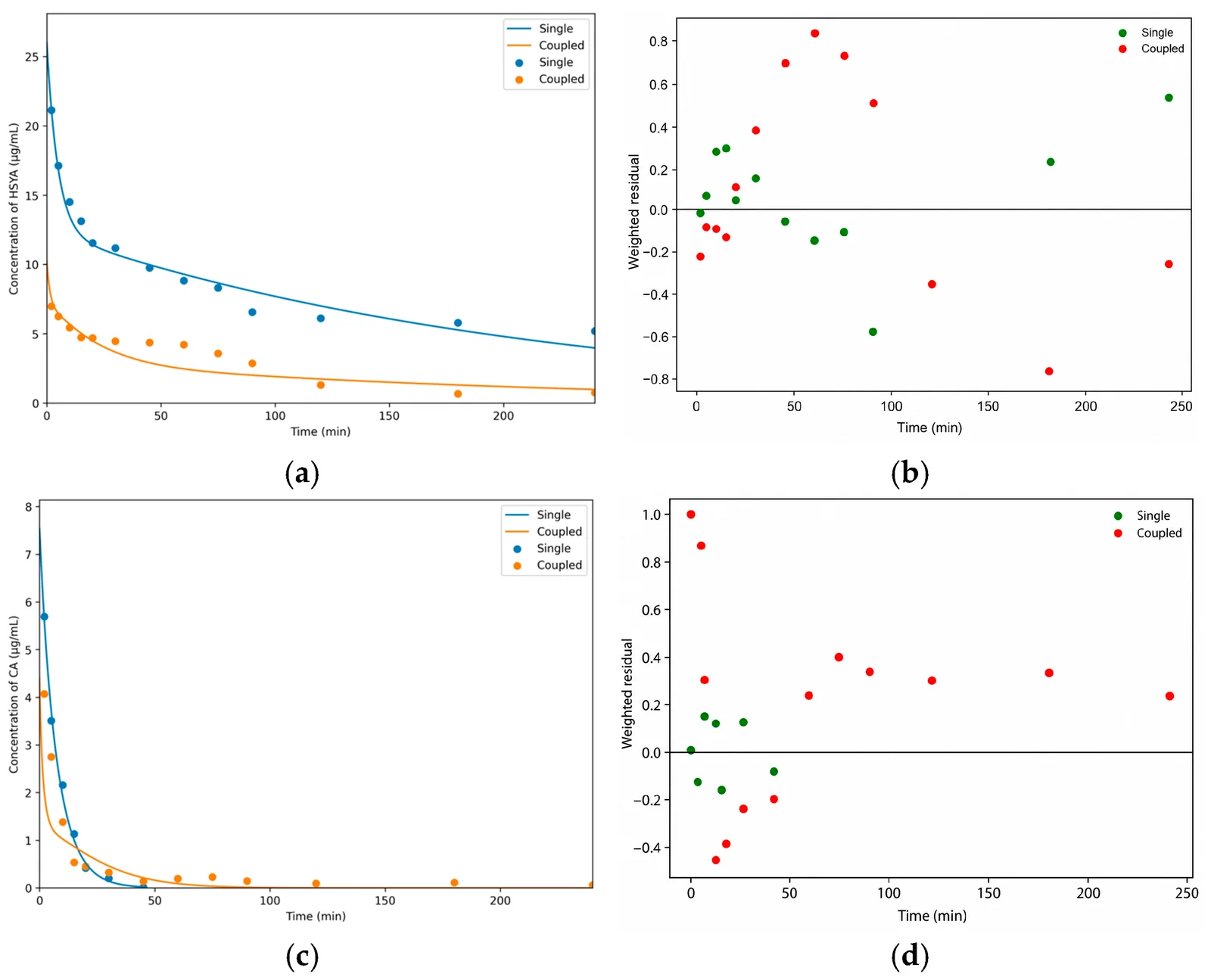
Fitting results and weighted residual distributions for the coupled PK model: (**a**) HSYA fitting results; (**b**) HSYA weighted residual distribution; (**c**) CA fitting results; (**d**) CA weighted residual distribution.

Figure 4 shows the results of the PK model fitting based on sample means and the corresponding weighted residual distributions. The model fitting curves for HSYA and CA generally align with the measured concentration-time profiles; the *R*^2^ values for HSYA alone and in combination were 0.97 and 0.83, respectively, while those for CA alone and in combination were 0.99 and 0.86, respectively. The weighted residuals are primarily distributed around 0. The results of the normality test indicate that the weighted residuals for both HSYA and CA do not deviate significantly from a normal distribution (*p* > 0.2); the test results for the mean residuals show that there is no significant difference between the mean residuals of both compounds and zero (*p* > 0.1). Furthermore, no obvious regular patterns were observed in the distribution of residuals over time or with respect to predicted concentrations. The above results indicate that the model residuals are generally randomly distributed, with no obvious systematic bias detected, suggesting that the model can adequately describe the concentration-time variations in HSYA and CA under the current experimental conditions.

**Figure 5:**
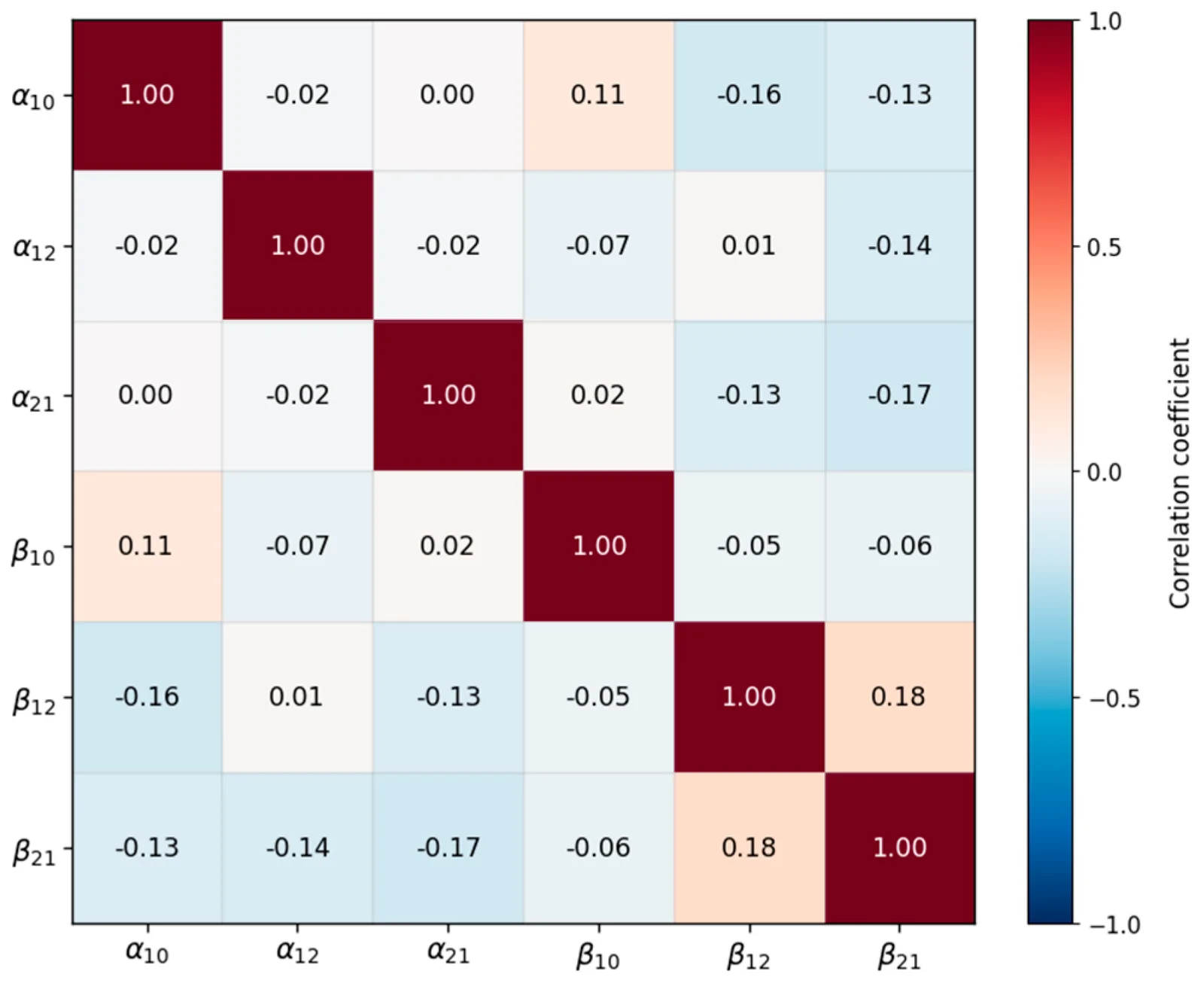
Correlation heatmap of coupling parameters based on Bootstrap sampling. Blue indicates negative correlations, red positive correlations, and darker colors indicate stronger correlations.

Based on this, the Bootstrap method was used to further evaluate the uncertainty in the estimates of the coupling parameters and the correlations among the parameters. The final parameter and their 95% Bootstrap confidence intervals are shown in Table 3, and the heatmap of the parameter correlation matrix is shown in Figure 5. There are some differences in the widths of the confidence intervals for the various coupling parameters, reflecting differences in the degree of uncertainty in the estimates of each parameter. The Bootstrap 95% confidence intervals for all six coupling parameters exclude zero, indicating that, given the current data, model structure, and parameter constraints, the estimates for each coupling parameter are supported by non-zero values.

The parameter correlation matrix further shows that the correlation coefficients among the coupling parameters are generally low, and no significant strong correlations were observed. This indicates that the interdependence among parameter estimates is limited, and no obvious estimation instability resulting from high parameter correlations was observed. Based on a comprehensive analysis of structural identifiability, Bootstrap confidence intervals, and parameter correlation, the coupling parameters in the current model exhibit good estimability and numerical stability. The above results provide quantitative evidence for the existence of model-level PK interactions between HSYA and CA. However, this does not directly equate to pharmacological synergy, and its specific biological significance still requires interpretation in conjunction with parameter directions and experimental results.

PK indicators (e.g., apparent volume of distribution) were also computed for each compound under monotherapy and co-administration. For example, the apparent volume of distribution for HSYA changed from 36.1306 ± 5.2577 mL under monotherapy conditions to 40.1532 ± 3.4512 mL when co-administered, whereas the corresponding values for CA were 222.1013 ± 54.76421 mL and 229.4722 ± 16.8407 mL, respectively. Other pharmacokinetic indicators are shown in Table 4.

Intergroup comparisons of pharmacokinetic parameters were performed between the single-component administration group and the combination therapy group. For HSYA and CA, the aforementioned five PK parameters were defined as a multiple-test family. The Holm-Bonferroni method was used to correct the raw *p*-values to control the overall Type I error rate resulting from multiple comparisons, and the results are summarized in Table 5. The results show that, after Holm-Bonferroni correction, statistically significant differences in *AUC*_0-*t*_, *AUC*_0-*∞*_ and CL for HSYA and CA still existed between the monotherapy and combination therapy groups, whereas the differences in *MRT*_0-*t*_ and *MRT*_0-*∞*_ were not statistically significant.

### 3.3. Coupled PK-PD Model

Under the conditions where the basic PD parameters are fixed and the output of the coupled PK model is used as a known input, a structural identifiability analysis was performed on the parameters *α* and *β* in the combined pharmacodynamic model. The results show that *E*_1_(*t*) and *E*_2_(*t*) do not follow a constant proportional relationship and are linearly independent within the model analysis interval. Therefore, no two distinct parameter combinations can produce exactly the same combined pharmacodynamic output. Accordingly, both *α* and *β* can be uniquely determined within the current hierarchical estimation framework and exhibit global structural identifiability.

**Figure 6:**
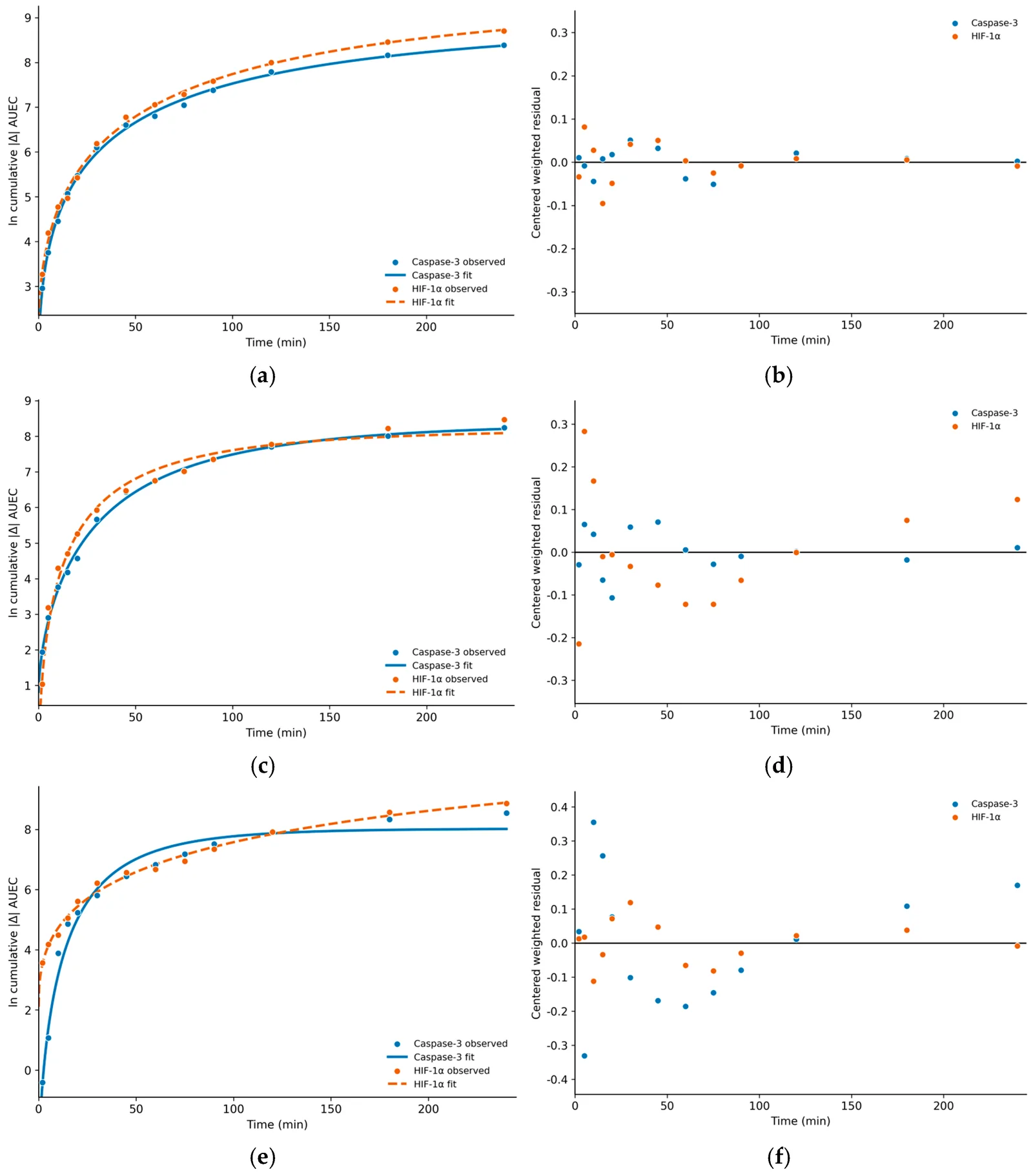
Results of PD model fitting for Caspase-3 and HIF-1α under HSYA, CA, and combined administration conditions, along with the distributions of weighted residuals. (**a**) Model fitting results for Caspase-3 and HIF-1α under HSYA monotherapy; (**b**) Distribution of weighted residuals for Caspase-3 and HIF-1α under HSYA monotherapy; (**c**) Model fitting results for Caspase-3 and HIF-1α under CA monotherapy; (**d**) Weighted residual distribution for Caspase-3 and HIF-1α under CA monotherapy; (**e**) Model fitting results for Caspase-3 and HIF-1α under combination therapy; (**f**) Weighted residual distribution for Caspase-3 and HIF-1α under combination therapy.

Figure 6 shows the fitting results and residuals for the PD data. Overall, the fitted curves of each model are consistent with the trends of the corresponding experimental observations, and *R*^2^ for each fit is greater than 0.97. The residuals for each group are primarily distributed around 0; normality tests showed no significant deviation from a normal distribution (*p* > 0.6), and tests of the mean residuals also indicated no significant difference between the mean residuals of each group and 0 (*p* > 0.8). Furthermore, the residuals did not exhibit any obvious regular patterns over time or relative to the model predictions. These results indicate that no significant systematic bias was observed during the fitting of the coupled PK-PD model and that the model adequately describes the effect-time relationship under the current experimental conditions.

The Bootstrap method was further employed to evaluate the uncertainty in the parameter estimates of the coupled PK-PD model. The final estimates of the single-component PD parameters and the coupled PK-PD parameters, along with their 95% Bootstrap confidence intervals, are shown in Table 6. The 95% confidence intervals for both coupled parameters *α* and *β* do not include 0, indicating that, given the current data, model structure, and parameter constraints, both *α* and *β* are supported by non-zero parameter estimates. The correlation coefficient between *α* and *β* obtained from Bootstrap resampling is 0.25, showing no obvious strong correlation, suggesting that the interdependence between the two parameter estimates is limited.

Based on the results of structural identifiability, fit and residual diagnostics, and Bootstrap uncertainty analysis, *α* and *β* exhibit good estimability and numerical stability in the current hierarchical coupled PK-PD model. It should be noted that *α* and *β* are coupling parameters derived from the model; their estimates are used to quantify the model-level contributions of the two components to the therapeutic response under co-administration conditions and do not directly equate to experimentally validated pharmacological synergism.

### 3.4. Model Predictability, Robustness and Sensitivity Analysis

#### 3.4.1. Predictive Performance Analysis

To further evaluate the predictive capabilities of the coupled PK and PK-PD models for data not used in parameter estimation, pointwise leave-one-out cross-validation was used to assess their internal predictive performance. The evaluation metrics were the cross-validation coefficient of determination (*Q*^2^) and the root mean square error (RMSE), calculated using the following formulas

(6) $$Q^{2}=1-\frac{\sum_{i=1}^{n}{\left(y_{i}-{\hat{y}}_{-i}\right)}^{2}}{\sum_{i=1}^{n}{\left(y_{i}-\bar{y}\right)}^{2}}$$

(7) $$RMSE=\sqrt{\frac{1}{n}\sum_{i=1}^{n}{\left(y_{i}-{\hat{y}}_{-i}\right)}^{2}}$$

where *y_i_* represents the actual observation, *ŷ*_−*i*_ denotes the cross-validation prediction obtained after holding out the *i*-th observation, *ȳ* is the mean of the actual observations, and *n* is the number of observations included in the evaluation. The closer *Q*^2^ is to 1 and the smaller the RMSE, the better the model’s predictive performance.

The results showed that the cross-validation coefficients of determination (*Q*^2^) for the coupled PK models of HSYA and CA were 0.878 and 0.906, respectively, with RMSE values of 0.668 and 0.364, respectively. This indicates that the coupled PK models for both components exhibited good predictive ability for time points not used in parameter estimation. For the coupled PK-PD models, the *Q*^2^ values for Caspase-3 and HIF-1α were 0.942 and 0.991, respectively, with RMSE values of 0.666 and 0.165, respectively. Since the PK-PD models were estimated using log-transformed AUEC values, their RMSE values were calculated on the log (AUEC) scale. Overall, the cross-validation *Q*^2^ values for all models were greater than 0.85, with low prediction errors, indicating that the established coupled PK and PK-PD models exhibited good internal predictive performance within the current group-mean time-series data.

#### 3.4.2. Robustness Analysis

To evaluate the sensitivity of the particle swarm optimization algorithm to random initial conditions and the numerical stability of the model parameter estimates, parameter optimization was repeated 100 times under different random initial conditions, while keeping the model structure, parameter constraints, and objective function unchanged. An optimization run was considered to have achieved stable convergence when the relative difference between its resulting objective function value and the minimum objective function value among the 100 runs did not exceed 5%. The coefficient of variation (CV) was used to assess the dispersion of the objective function values and the key coupling parameters obtained from the repeated optimizations.

(8) $$CV=\frac{s}{\left|\bar{x}\right|}\times 100\%$$

where $\bar{x}$ represents the mean of the objective function values or parameter estimates obtained from 100 repeated optimizations, and *s* is the corresponding standard deviation. A smaller CV indicates greater stability of the optimization results under different random initial conditions.

The repeated optimization results showed that 94 out of 100 runs met the stable convergence criterion, yielding a stable convergence rate of 94%. The CV of the objective function values was 2.7%, and the CV of the major coupling parameters ranged from 3.6% to 12.8%. Under different random initial conditions, the signs and overall directional trends of all key coupling parameters remained consistent, with no obvious parameter drift observed. These results demonstrate that the adopted hierarchical particle swarm optimization method exhibits good numerical stability and robustness with respect to random initial conditions.

#### 3.4.3. Sensitivity Analysis

New coupling parameters were added in the model. Local sensitivity analyses with single variable changes were carried out. The analyses targeted coupled PK models and coupled PK-PD models. The work aimed to check how new coupling parameters affect model outputs. Final calculated values of all parameters served as fixed baseline data. Each parameter received a separate adjustment of plus or minus 10%. All other parameter values stayed the same during the adjustment process. Six parameters were checked for the coupled PK model. These parameters include *α*_10_, *α*_12_, *α*_21_, *β*_10_, *β*_12_ and *β*_21_. AUC values within 0–240 min were recorded. The relative variation in these AUC values judged parameter sensitivity. Two pharmacodynamic coupling parameters α and β were tested for the coupled PK-PD model. Sensitivity results came from varied relative contribution coefficients of HSYA and CA.

HSYA AUC values showed obvious responses to changes in *α*_10_. A 10% drop in *α*_10_ brought a 3.13% rise in AUC. A 10% rise in*α*_10_ brought a 3.06% drop in AUC. Adjustments to *α*_12_ created around 0.26% fluctuation in AUC values. *α*_21_ caused AUC fluctuations lower than 0.04%. CA AUC values had the most obvious response to *β*_10_. A 10% decrease in *β*_10_ led to a 7.44% AUC increase. A 10% increase in *β*_10_ led to a 6.71% AUC decrease. *β*_12_ and *β*_21_ both triggered AUC changes below 0.2%. Coupling parameters controlling the *k*_10_ elimination process alter drug exposure levels greatly. Coupling parameters for intercompartmental distribution produce weaker effects on drug exposure.

For the coupled PK-PD model, ±10% perturbations were separately applied to the two pharmacodynamic coupling parameters. The resulting relative contribution coefficients showed limited variation, and the overall contribution pattern of HSYA and CA remained stable for both Caspase-3 and HIF-1α. These results indicate that moderate perturbations of the PD coupling parameters did not materially alter the interpretation of the relative pharmacodynamic contributions.

## 4. Discussion

The pathological mechanisms underlying CIRI are complex and involve multiple temporally evolving processes, including oxidative stress, neuroinflammation, ferroptosis, vascular injury, and other interconnected pathways [17,18,19,20,21,22,23]. Accordingly, multi-component and multi-target interventions based on natural products and TCM have attracted increasing attention in the treatment of cerebral ischemic injury [8,24,25]. Due to the multi-component and multi-target nature of TCM, there is currently a lack of analytical methods that can simultaneously quantitatively analyze the pharmacokinetic interactions between active components and their specific efficacy contributions [26]. To overcome this limitation, a coupled PK model was proposed. The results indicate that co-administration was associated with changes in the pharmacokinetic profiles of HSYA and CA, while the coupled PK-PD model adequately characterized the dynamic relationship between drug exposure and pharmacodynamic responses. Furthermore, the contribution coefficient can be used to analyze the respective pharmacodynamic contributions of the two components.

The coupled PK model exhibited good performance in fitting the pharmacokinetic profiles of HSYA and CA after co-administration. The *R*^2^ were 0.83 and 0.86 for HSYA and CA, respectively, confirming that this model reliably describes the in vivo concentration-time characteristics of the two components under combined administration. In addition, conditional structural identifiability analysis demonstrated that the six newly introduced PK coupling parameters were globally identifiable when the baseline PK parameters were fixed. Their Bootstrap 95% confidence intervals excluded zero, and no strong correlations among the coupling parameters were observed. However, the limited availability of measurable CA concentrations at later time points may reduce the precision of elimination-related parameter estimates. Although the single-compound PK model has an analytical solution, this cannot compensate for limited terminal-phase observations. Therefore, the CA elimination parameters should be interpreted cautiously, and extended sampling may improve their reliability in future studies.

Compared with single administration, co-administration was associated with decreased *AUC*_0-*t*_, *AUC*_0-∞_ and increased *CL* for both HSYA and CA after Holm-Bonferroni correction, whereas *MRT*_0-*t*_ and *MRT*_0-∞_ did not differ significantly. These results indicate that the principal pharmacokinetic changes were related to systemic exposure and clearance rather than residence time. Because HSYA and CA were administered intravenously, these differences are more appropriately considered in relation to distribution and elimination processes rather than absorption. CYP450-mediated metabolism provides one biologically plausible explanation for the observed changes [27]. Previous studies have shown that HSYA can modulate the activities of several CYP isoenzymes [28], while CA may also influence CYP-mediated drug metabolism [29]. Nevertheless, these mechanisms were not directly evaluated in the present study and therefore remain hypotheses requiring experimental verification. Furthermore, the limited number of measurable CA concentrations during the terminal phase may reduce the precision of elimination-related parameter estimates, and extended sampling would improve their reliability.

For the pharmacodynamic component, the effect-compartment and indirect-response structures were incorporated to account for the temporal relationship between plasma exposure and the observed Caspase-3 and HIF-1α responses. The resulting models showed good fitting performance, with *R*^2^ values above 0.97 and no evident systematic pattern in the residuals. The two PD coupling parameters were also globally identifiable within the hierarchical estimation framework, and their Bootstrap 95% confidence intervals excluded zero. The model-derived contribution coefficients indicated comparable contributions of HSYA and CA to the Caspase-3 response, with Bootstrap 95% confidence intervals of 0.4285–0.5104 and 0.4896–0.5715, respectively. For HIF-1α, the corresponding intervals were 0.4375–0.5640 and 0.4360–0.5625, indicating similarly balanced relative contributions of the two components under the current model. Previous mechanistic studies concerning SIRT1/NF-κB, HIF-1α/NOX2, JAK2/STAT3, oxidative stress, and neuroinflammation may provide biological context for these differences [8,30,31,32,33,34]. However, the contribution coefficients are model-derived relative weights and should not be interpreted as direct evidence of pharmacological synergy or as definitive evidence of hierarchical functional roles between the two compounds. Indeed, the current results already explicitly distinguish these model-derived contributions from experimentally validated pharmacological synergism.

The present framework is also consistent with the broader development of quantitative PK-PD approaches for multi-component TCM systems. Previous studies have applied coupled PK-PD modeling to characterize component-dependent effects [35], integrated polypharmacokinetic-pharmacodynamic strategies to evaluate active substances in Astragali Radix-Carthami Flos combinations [36], and conventional PK-PD models to other herb-pair systems [37]. Recent technical guidelines have further emphasized standardized non-clinical pharmacological evaluation of TCM formulae [38]. In addition, approaches combining microdialysis with LC-MS-based PK-PD analysis illustrate the potential of site-specific concentration measurements for linking tissue exposure with pharmacodynamic responses [39]. These developments provide a methodological context for extending multi-component PK-PD analysis beyond plasma-based observations.

The additional model evaluation further supports the numerical reliability of the proposed framework. Leave-one-time-point-out cross-validation yielded *Q*^2^ values of 0.878 and 0.906 for the HSYA and CA coupled PK models and 0.942 and 0.991 for the Caspase-3 and HIF-1α PK-PD models, respectively, indicating satisfactory internal predictive performance within the current dataset. Repeated optimization from 100 different random initial conditions resulted in a stable convergence rate of 94%, with limited variability in the objective function and major coupling parameters. In addition, local sensitivity analysis showed that the exposure profiles were more sensitive to coupling parameters associated with the elimination process than to those associated with intercompartmental distribution, whereas the relative ordering of the pharmacodynamic contributions of HSYA and CA remained unchanged under ±10% perturbation. These analyses indicate that the hierarchical estimation strategy provides numerically stable estimates under the current experimental setting. Such sequential and algorithm-assisted optimization strategies are particularly useful in complex pharmacometric models, where high-dimensional parameter estimation and model selection may otherwise increase numerical instability and the risk of convergence to suboptimal solutions [40]. Importantly, the hierarchical strategy itself should be regarded as a means of reducing the dimensionality of simultaneous optimization, whereas structural identifiability is established separately by the SIAN analysis.

It should also be recognized that comparison with the corresponding conventional non-coupled models did not demonstrate a clear statistical advantage of the coupled models according to AICc, BIC and likelihood-ratio testing (see File S4 and Table S1). Therefore, the value of introducing the coupling terms lies primarily in providing an interpretable quantitative representation of potential component-dependent changes rather than in demonstrating superior overall goodness of fit. This distinction is important because the additional coupling parameters should not themselves be taken as statistical proof that biological interactions exist.

Several limitations should be considered. First, the linear coupling functions represent local approximations within the investigated dose and concentration ranges and may not capture nonlinear, saturable, or competitive interactions under other exposure conditions. Second, only six independent animals were included in each treatment group, and the cross-validation and Bootstrap analyses provide internal rather than external validation. Third, terminal-phase CA observations were relatively limited, potentially affecting the precision of elimination-related parameters. Fourth, only Caspase-3 and HIF-1α were used as pharmacodynamic endpoints. Future studies using larger independent datasets, extended pharmacokinetic sampling, additional pharmacodynamic biomarkers, and potentially nonlinear coupling structures will be required to further evaluate the generalizability and biological interpretation of the proposed framework. In parallel, data-driven and artificial intelligence-assisted strategies may provide complementary approaches for optimizing complex TCM combinations and informing model refinement [41].

## 5. Conclusions

In this study, HSYA and CA were investigated using a coupled PK-PD model in a rat model of CIRI. The results indicated pharmacokinetic interactions between HSYA and CA following co-administration. This model enabled the quantitative evaluation of the PK profiles, PD effects, and inter-component interactions of HSYA and CA following both single and combined administration. The developed Coupled PK and Coupled PK-PD models adequately characterized the in vivo kinetic behavior and pharmacodynamic responses of the combined regimen. Furthermore, the normalized model-derived relative contribution weights characterized the relative contributions of HSYA and CA across different pharmacodynamic endpoints. The coupled PK-PD model established in this study integrates pharmacokinetic interactions, dynamic pharmacodynamic responses, and constituent-specific contribution analysis into a unified quantitative model, providing a novel analytical strategy for PK-PD studies of multi-component TCM.

## Figures and Tables

**Table 1:** Experimental data for HSYA and CA ($\bar{x}$ ± s, *n* = 6).

Time	HSYA	CA	HSYA (Coupled)	CA (Coupled)
2	21.1264 ± 3.0406	5.6908 ± 1.4020	6.9800 ± 0.1054	4.0733 ± 0.2523
5	17.1246 ± 0.5567	3.5060 ± 0.2516	6.2523 ± 0.4691	2.7501 ± 0.4210
10	14.5097 ± 0.5790	2.1608 ± 0.1492	5.4542 ± 0.1479	1.3827 ± 0.1778
15	13.1308 ± 1.5077	1.1335 ± 0.1179	4.7370 ± 0.0985	0.5341 ± 0.2087
20	11.5480 ± 0.5504	0.4193 ± 0.1094	4.7021 ± 0.1435	0.4526 ± 0.1444
30	11.1915 ± 0.4854	0.2025 ± 0.0535	4.4622 ± 0.1354	0.3240 ± 0.0136
45	9.7647 ± 0.5903	0.0117 ± 0.0037	4.3702 ± 0.1302	0.1399 ± 0.0148
60	8.8318 ± 0.4156	−	4.2137 ± 0.0292	0.1985 ± 0.0830
75	8.3228 ± 0.7008	−	3.5896 ± 0.0853	0.2279 ± 0.0914
90	6.5590 ± 1.3592	−	2.8656 ± 0.0885	0.1434 ± 0.0611
120	6.1164 ± 0.9672	−	1.3100 ± 0.0333	0.0982 ± 0.0290
180	5.7964 ± 0.3543	−	0.6682 ± 0.0348	0.1150 ± 0.0248
240	5.1938 ± 0.8408	−	0.7532 ± 0.1069	0.0601 ± 0.0191

“−” indicates concentrations that were not measured.

**Table 2:** Experimental data for Caspase-3 and HIF-1α ($\bar{x}$ ± s, *n* = 6).

Time	Caspase-3/pmol∙L^−1^				HIF-1α/pg∙mL^−1^			
	MCAO/R(M)	M + HSYA	M + CA	M + HSYA + CA	MCAO/R(M)	M + HSYA	M + CA	M + HSYA + CA
2	82.1125 ± 2.3973	72.5092 ± 2.5736	78.6393 ± 2.8125	82.4462 ± 3.3148	120.6840 ± 4.6372	133.8512 ± 3.6245	119.2750 ± 3.8237	103.0230 ± 3.4842
5	80.2506 ± 1.9998	86.3453 ± 2.3256	76.1931 ± 3.9033	79.0867 ± 3.0450	119.4557 ± 6.7661	132.8083 ± 4.9806	132.2664 ± 4.0173	121.5820 ± 4.1794
10	90.3659 ± 2.2531	79.1451 ± 1.9685	84.4895 ± 2.5103	73.2196 ± 2.1970	129.4051 ± 5.3123	136.9003 ± 4.1960	136.2139 ± 3.3549	121.9770 ± 3.8999
15	94.6418 ± 2.5167	76.5529 ± 2.1684	91.6544 ± 2.0550	79.9001 ± 3.4049	130.0000 ± 5.9484	127.2038 ± 6.1783	138.0275 ± 4.7789	110.4700 ± 4.0541
20	92.5953 ± 3.2693	79.3312 ± 1.7322	82.9797 ± 2.1749	83.8084 ± 3.9710	146.0429 ± 4.7171	115.2192 ± 3.2084	121.1802 ± 4.1769	118.7810 ± 6.1689
30	98.8469 ± 3.3094	70.4013 ± 2.0044	69.9154 ± 2.9315	78.5301 ± 2.4655	139.1065 ± 7.0631	118.3671 ± 4.6356	127.3483 ± 5.1055	120.7800 ± 4.6264
45	86.2700 ± 3.4995	75.7749 ± 3.4922	70.5397 ± 1.7530	67.3755 ± 2.4941	144.8555 ± 3.7561	113.4128 ± 3.4670	120.3613 ± 4.7042	135.3810 ± 3.6384
60	89.9692 ± 2.1121	79.2005 ± 3.4818	73.5716 ± 2.6289	69.5756 ± 2.0909	133.6946 ± 8.1249	127.3001 ± 4.5822	130.0385 ± 4.9592	134.5690 ± 4.2743
75	94.3942 ± 5.0088	71.3792 ± 3.3704	76.4745 ± 1.8608	62.4677 ± 3.2168	147.4711 ± 3.5149	113.5573 ± 4.3339	116.8931 ± 6.5271	115.4790 ± 6.0540
90	106.7174 ± 3.1278	69.841 ± 3.3316	70.9425 ± 1.7841	69.3127 ± 2.7667	145.2408 ± 4.9178	113.0395 ± 5.8986	116.6811 ± 4.3674	109.2700 ± 6.6139
120	92.5338 ± 3.0860	74.787 ± 2.5498	81.7743 ± 3.7633	66.1516 ± 3.1960	159.8627 ± 4.4848	124.7013 ± 5.2942	134.3256 ± 5.6021	117.0330 ± 5.2693
180	98.3518 ± 2.0925	80.0031 ± 3.4393	83.4871 ± 3.5110	79.1621 ± 2.1341	151.5800 ± 4.0931	128.4827 ± 6.1019	132.2519 ± 6.1765	109.4510 ± 6.1943
240	93.4210 ± 2.2117	82.7691 ± 2.5412	81.6820 ± 2.7291	79.9677 ± 2.2923	149.0727 ± 3.8420	128.6922 ± 6.9533	133.4562 ± 3.8419	130.8480 ± 3.9934

**Table 3:** Bootstrap 95% Confidence Intervals for Parameters of the HSYA and CA Coupled PK Models.

	Parameter	HSYA	CA
Single	*k*_10_	[0.0052, 0.0141]	[0.1023, 0.1697]
	*k*_12_	[0.0430, 0.1597]	[0.0062, 0.0164]
	*k*_21_	[0.0391, 0.1622]	[0.2098, 0.5502]
Coupled	*α*_10_ (*β*_10_)	[0.0052, 0.0138] (*α*_10_)	[0.0434, 0.0725] (*β*_10_)
	*α*_12_ (*β*_12_)	[0.0016, 0.0951] (*α*_12_)	[0.0192, 0.0400] (*β*_12_)
	*α*_21_ (*β*_21_)	[−0.1409, −0.0112] (*α*_21_)	[0.0480, 0.1838] (*β*_21_)

**Table 4:** PK indicators for HSYA and CA ($\bar{x}$ ± s, *n* = 6).

Parameter	HSYA (Single)	HSYA (Coupled)	CA (Single)	CA (Coupled)
*AUC*_0-*t*_*/*μg ∙ mL^−1^ ∙ h	31.3446 ± 5.1594	8.8070 ± 2.1166	0.9350 ± 0.0914	0.6359 ± 0.1427
*AUC*_0-∞_*/*μg ∙ mL^−1^ ∙ h	52.4944 ± 9.8982	12.2501 ± 4.5530	1.2350 ± 0.1142	1.0359 ± 0.0427
*MRT*_0-*t*_/h	1.6238 ± 0.321	1.4023 ± 0.222	0.2421 ± 0.0298	0.2166 ± 0.0139
*MRT*_0-∞_/h	4.4255 ± 1.3699	3.1162 ± 0.2761	0.3221 ± 0.168	0.3466 ± 0.109
*CL/*mL ∙ h^−1^	14.2872 ± 3.0498	61.2239 ± 12.7400	1283.4085 ± 123.1283	1887.1955 ± 127.3448

**Table 5:** Comparison of PK parameters under monotherapy and combination therapy conditions, and results of multiple comparison corrections (*n* = 6).

Parameter	HSYA	CA
*AUC*_0-*t*_*/*μg ∙ mL^−1^ ∙ h	0.000162	0.00877
*AUC*_0-∞_*/*μg ∙ mL^−1^ ∙ h	0.000165	0.0135
*MRT*_0-*t*_/h	0.198	0.691
*MRT*_0-∞_/h	0.132	0.772
*CL*/mL ∙ h^−1^	0.000544	0.000407

**Table 6:** Parameter estimates and 95% Bootstrap confidence intervals for the PD model of Caspase-3 and HIF-1α.

Parameter	Caspase-3		HIF-1α	
	HSYA	CA	HSYA	CA
*k*_e_	[0.2990, 0.3326]	[0.4401, 0.5941]	[0.2987, 0.3270]	[0.4073, 0.6950]
*k*_in_	[0.0966, 0.1311]	[0.1421, 0.1622]	[0.0874, 0.1167]	[0.1435, 0.1892]
*k*_out_	[0.0235, 0.0365]	[0.0172, 0.0203]	[0.0200, 0.0305]	[0.0173, 0.0242]
*E*_max_	[1.2125, 1.4738]	[0.7760, 0.9746]	[1.1959, 1.4164]	[1.1978, 1.7138]
*EC*_50_	[1.4508, 2.0711]	[2.7809, 3.5117]	[1.5876, 2.0620]	[3.3690, 4.9689]
*α*(*β*)	[0.4720, 0.5742]	[0.5407, 0.6417]	[0.4782, 0.6388]	[0.4800, 0.6337]
*η*	[0.4285, 0.5104]	[0.4896, 0.5715]	[0.4375, 0.5640]	[0.4360, 0.5625]

## Data Availability

The original contributions presented in this study are included in the article/Supplementary Materials. Further inquiries can be directed to the corresponding author.

## Supplementary Materials

The following supporting information can be downloaded at https://www.mdpi.com/article/10.3390/biology15171543/s1: File S1: Coupled PK Model; File S2: Coupled PK-PD Model; File S3: Hierarchical Optimization Algorithm; File S4: Comparison between Conventional and Coupled PK–PD models; Table S1. Comparison between conventional and coupled PK/PK–PD models.

## References

[1] Csajka C., Verotta D. (2006). Pharmacokinetic-pharmacodynamic modelling: History and perspectives. J. Pharmacokinet. Pharmacodyn..

[2] Qu C., Xu D.Q., Yue S.J., Shen L.F., Zhou G.S., Chen Y.Y., Wang X.P., Bai J.Q., Liu F., Tang Y.P. (2020). Pharmacodynamics and of Danshen in isoproterenol-induced acute myocardial ischemic injury combined with Honghua. J. Ethnopharmacol..

[3] Long W., Zhang S.C., Wen L., Mu L., Yang F., Chen G. (2014). In vivo distribution and pharmacokinetics of multiple active components from Danshen and Sanqi and their combination via inner ear administration. J. Ethnopharmacol..

[4] Gong L., Miao Z., Zhang L., Shi B., Xiao Z., Qiu P., Liu M., Zou W. (2022). Pharmacokinetics and pharmacodynamics of bioactive compounds in Penyanqing preparation in THP-1 inflammatory cells induced by Lipopolysaccharide. BMC Complement. Med. Ther..

[5] Lai Y.W., Liu Z.W., Lin M.H., Yang C.C., Chu C.Y., Chung C.H., Lin C.W. (2024). Melatonin increases Olaparib sensitivity and suppresses cancer-associated fibroblast infiltration via suppressing the LAMB3-CXCL2 axis in TNBC. Pharmacol. Res..

[6] Rasoanaivo P., Wright C.W., Willcox M.L., Gilbert B. (2011). Whole plant extracts versus single compounds for the treatment of malaria: Synergy and positive interactions. Malar. J..

[7] Yu L., Liu Z., He W., Chen H., Lai Z., Duan Y., Cao X., Tao J., Xu C., Zhang Q. (2020). Hydroxysafflor Yellow A Confers Neuroprotection from Focal Cerebral Ischemia by Modulating the Crosstalk Between JAK2/STAT3 and SOCS3 Signaling Pathways. Cell Mol. Neurobiol..

[8] Fangma Y., Zhou H., Shao C., Yu L., Yang J., Wan H., He Y. (2021). Hydroxysafflor Yellow A and Anhydrosafflor Yellow B Protect Against Cerebral Ischemia/Reperfusion Injury by Attenuating Oxidative Stress and Apoptosis via the Silent Information Regulator 1 Signaling Pathway. Front. Pharmacol..

[9] Yang X., Pan Y., Cai L., Wang W., Zhai X., Zhang Y., Wu Q., Chen J., Zhang C., Wang Y. (2024). Calycosin Ameliorates Neuroinflammation via TLR4-Mediated Signal Following Cerebral Ischemia/Reperfusion Injury in vivo and in vitro. J. Inflamm. Res..

[10] Chen Q., Wan J., Zhang Y., He Y., Bao Y., Yu L., Yang J. (2022). Pharmacokinetic-pharmacodynamic modeling analysis for hydroxysafflor yellow A-calycosin in compatibility in normal and cerebral ischemic rats: A comparative study. BioMed Pharmacother..

[11] Longa E.Z., Weinstein P.R., Carlson S., Cummins R. (1989). Reversible middle cerebral artery occlusion without craniectomy in rats. Stroke.

[12] Li-Weber M. (2013). Targeting apoptosis pathways in cancer by Chinese medicine. Cancer Lett..

[13] Li W., Liu X., Liu Z., Xing Q., Liu R., Wu Q., Hu Y., Zhang J. (2024). The signaling pathways of selected traditional Chinese medicine prescriptions and their metabolites in the treatment of diabetic cardiomyopathy: A review. Front. Pharmacol..

[14] Holz M., Fahr A. (2001). Compartment modeling. Adv. Drug Deliv. Rev..

[15] Pan S., Zhou J., Zhou S., Huang Z., Meng J. (2020). Pharmacokinetic-pharmacodynamic modeling for Moutan Cortex/Moutan Cortex charcoal and the contributions of the chemical component using support vector regression with particle swarm optimization. RSC Adv..

[16] Hong H., Ovchinnikov A., Pogudin G., Yap C. (2019). SIAN: Software for structural identifiability analysis of ODE models. Bioinformatics.

[17] Qin C., Yang S., Chu Y.H., Zhang H., Pang X.W., Chen L., Zhou L.Q., Chen M., Tian D.S., Wang W. (2022). Signaling pathways involved in ischemic stroke: Molecular mechanisms and therapeutic interventions. Signal Transduct. Target Ther..

[18] Yuan Y., Sheng P., Ma B., Xue B., Shen M., Zhang L., Li D., Hou J., Ren J., Liu J. (2023). Elucidation of the mechanism of Yiqi Tongluo Granule against cerebral ischemia/reperfusion injury based on a combined strategy of network pharmacology, multi-omics and molecular biology. Phytomedicine.

[19] Shi F.N., Yu Z.D., Wang X.N., Zhang Z.C. (2026). Time-Dependent Neuroprotection and Precision Interventions in Acute Ischemic Stroke in the Reperfusion Era. Chin. Med. Sci. J..

[20] Xu L., Li C., Zhao C., Wang Z., Zhang Z., Shu X., Cao X., Xia S., Bao X., Shao P. (2025). Shionone protects cerebral ischemic injury through alleviating microglia-mediated neuroinflammation. Chin. J. Nat. Med..

[21] Liu K., Wang L., Pang T. (2025). Research progress of small-molecule natural medicines for the treatment of ischemic stroke. Chin. J. Nat. Med..

[22] Wang Y., Lan X., Liu N., Ma L., Du J., Wei W., Hai D., Wu J., Yu J., Liu Y. (2024). Traditional Chinese medicines derived natural inhibitors of ferroptosis on ischemic stroke. Chin. J. Nat. Med..

[23] Yang Y., Che Y., Fang M., Yao X., Zhou D., Wang F., Chen G., Liang D., Li N., Hou Y. (2024). Reynosin protects neuronal cells from microglial neuroinflammation by suppressing NLRP3 inflammasome activation mediated by NADPH oxidase. Chin. J. Nat. Med..

[24] Zhao C., Bai X., Ding Y., Zeng A., Wen A., Fu Q., Wang J. (2026). The role of salvianolic acid B and benzoylpaeoniflorin in enhancing angiogenesis through Nrf2/HO-1/VEGFA signaling axis in ischemic stroke recovery. Pharm. Biol..

[25] Chen J., Wang Y., Wang S., Zhao X., Zhao L., Wang Y. (2022). Salvianolic acid B and ferulic acid synergistically promote angiogenesis in HUVECs and zebrafish via regulating VEGF signaling. J. Ethnopharmacol..

[26] Qi H., Chen S., Chen X., Ma Q., Shi J., Li X., Hou Y., Feng W., Zhu C., Liao S. (2025). Multi-dimensional PK-PD insights into Lianhua Qingwen’s formula compatibility. J. Ethnopharmacol..

[27] Tracy T.S., Chaudhry A.S., Prasad B., Thummel K.E., Schuetz E.G., Zhong X.B., Tien Y.C., Jeong H., Pan X., Shireman L.M. (2016). Interindividual Variability in Cytochrome P450-Mediated. Drug Metab. Dispos..

[28] Xu R.A., Xu Z.S., Ge R.S. (2014). Effects of hydroxysafflor yellow A on the activity and mRNA expression of four CYP isozymes in rats. J. Ethnopharmacol..

[29] Wu M.L., Lin Y.P., Wei Y.L., Du H.J., Ying X.Q., Tan W.Z., Tang B.E. (2020). Calycosin Influences the Metabolism of Five Probe Drugs in Rats. Drug Des. Dev. Ther..

[30] Gao H., Yang L., Shao Y. (2022). SIRT1/NF-κB pathway on neuronal apoptosis in rats with ischemic stroke. Cell Mol. Biol..

[31] Li Y., Liu X.T., Zhang P.L., Li Y.C., Sun M.R., Wang Y.T., Wang S.P., Yang H., Liu B.L., Wang M. (2022). Hydroxysafflor Yellow A Blocks HIF-1α Induction of NOX2 and Protects ZO-1 Protein in Cerebral Microvascular Endothelium. Antioxidants.

[32] Mahmoud A.M., Abd El-Twab S.M. (2017). Caffeic acid phenethyl ester protects the brain against hexavalent chromium toxicity by enhancing endogenous antioxidants and modulating the JAK/STAT signaling pathway. BioMed Pharmacother..

[33] Deng L., Wan H., Zhou H., Yu L., He Y. (2018). Protective effect of hydroxysafflor yellow A alone or in combination with acetylglutamine on cerebral ischemia reperfusion injury in rat: A PET study using (18)F-fuorodeoxyglucose. Eur. J. Pharmacol..

[34] Ahmed H.S. (2025). Neuropharmacological effects of calycosin: A translational review of molecular mechanisms and therapeutic applications. Naunyn Schmiedebergs Arch. Pharmacol..

[35] Sun J., Chen Q., Zhuang C., Li X., Yu L., Jin W. (2025). Mechanistic insights into synergistic effects using coupled PK-PD modeling. Sci. Rep..

[36] Zeng Q., Li C., Xu S., He Y. (2023). An integrated strategy to evaluate active substances of Astragali Radix-Carthami Flos combination on the treatment of cerebral ischemia reperfusion injury based on TQSM polypharmacokinetics and pharmacodynamics. J. Food Drug Anal..

[37] Wu E.H., Zhang J.H., Chen W.J., Wang Y.H., Yin H. (2025). Pharmacodynamics study and establishment of a PK-PD model for Epimedii Folium-Chuanxiong Rhizoma in treating osteoarthritis in rats. Zhongguo Zhong Yao ZA Zhi.

[38] Li W., Zhang F., Wang P., Zhang Y., Cao P., Ma Z., Yao H., Wang J., Yang H., Xu H. (2025). Technical guidelines for non-clinical pharmacological research of traditional Chinese medicine (TCM) formulae. Chin. Med..

[39] Zhang J., Cui X., Zhao S., Chang Z., Zhang J., Chen Y., Liu J., Sun G., Wang Y., Liu Y. (2024). Establishment of a pharmacokinetics and pharmacodynamics model of Schisandra lignans against hippocampal neurotransmitters in AD rats based on microdi-alysis liquid chromatography-mass spectrometry. Front. Pharmacol..

[40] Li X., Sale M., Nieforth K., Craig J., Wang F., Solit D., Feng K., Hu M., Bies R., Zhao L. (2024). PyDarwin machine learning algorithms application and comparison in nonlinear mixed-effect model selection and optimization. J. Pharmacokinet. Pharmacodyn..

[41] Tan S., Shao X., Zhang X., Dai Y., Zhang B., Cheng Y., Fan X. (2026). TCMNet: An AI-driven strategy for optimizing traditional Chinese medicine. Chin. Med..

