# Supplementary material for: Deciphering the Combined Effects of Hydroxysafflor Yellow A and Calycosin Through Coupled PK–PD Modeling
Source: Biology (Basel). 2026 Sep 4;15(17):1543. doi: 10.3390/biology15171543 (PMC13565704; doi:10.3390/biology15171543)
Supplement: Supplementary file 1 [file biology-15-01543-s001.zip › biology-4488035-supplementary.pdf]

File S1: Coupled PK Model

$$\begin{cases} \frac{dC_1^1}{dt} = -(k_{12}^1 + \beta_{12}C_1^2)C_1^1 + (k_{21}^1 + \beta_{21}C_2^2)C_2^1 - (k_{10}^1 + \beta_{10}C_1^2)C_1^1 \\ \frac{dC_2^1}{dt} = -(k_{21}^1 + \beta_{21}C_2^2)C_2^1 + (k_{12}^1 + \beta_{12}C_1^2)C_1^1 \\ \frac{dC_1^2}{dt} = -(k_{12}^2 + \alpha_{12}C_1^1)C_1^2 + (k_{21}^2 + \alpha_{21}C_2^1)C_2^2 - (k_{10}^2 + \alpha_{10}C_1^1)C_1^2 \\ \frac{dC_2^2}{dt} = -(k_{21}^2 + \alpha_{21}C_2^1)C_2^2 + (k_{12}^2 + \alpha_{12}C_1^1)C_1^2 \end{cases}$$

In this model, the parameters were defined as follows,

(1) C_j is the concentration of the ith component in compartment j; with the first and second components respectively corresponding to components A or B; and $j = 1$ (central compartment) or $j = 2$ (peripheral compartment).

(2) k_{10}^i is the first-order elimination rate constant of the ith component from the central compartment, k_{12}^i is the transfer rate constant of the ith component from the central to the peripheral compartment, and k_{21}^i is the transfer rate constant of the ith component from the peripheral compartment back to the central compartment.

(3) α_{kl} denotes the effect of component A on the pharmacokinetics of component B.

(4) β_{kl} denotes the effect of component B on the pharmacokinetics of component A.

Unlike traditional two-compartment models, this formulation accommodates inter-component modulation of ADME process. If α_{kl} and β_{kl} are zero, the model corresponds to the traditional case. The α_{kl} and β_{kl} parameters are additionally utilized to evaluate interactions between components. For instance, α_{10} is positive, implying that component A promotes the metabolism of component B in this scenario.

File S2: Coupled PK-PD Model /

PK Part

PD Part

$$
\begin{cases}
\frac{dC_1^1}{dt} = -\left(k_{12}^1 + \beta_{12}C_1^2\right)C_1^1 + \left(k_{21}^1 + \beta_{21}C_2^2\right)C_2^1 - \left(k_{10}^1 + \beta_{10}C_1^2\right)C_1^1 \\
\frac{dC_2^1}{dt} = -\left(k_{21}^1 + \beta_{21}C_2^2\right)C_2^1 + \left(k_{12}^1 + \beta_{12}C_1^2\right)C_1^1 \\
\frac{dC_1^2}{dt} = -(k_{12}^2 + \alpha_{12}C_1^1)C_1^2 + (k_{21}^2 + \alpha_{21}C_2^1)C_2^2 - (k_{10}^2 + \alpha_{10}C_1^1)C_1^2 \\
\frac{dC_2^2}{dt} = -(k_{21}^2 + \alpha_{21}C_2^1)C_2^2 + (k_{12}^2 + \alpha_{12}C_1^1)C_1^2 \\
\frac{dC_e^1}{dt} = k_e^1(C_1^1 - C_e^1) \\
\frac{dC_e^2}{dt} = k_e^2(C_1^2 - C_e^2) \\
\frac{dE_1}{dt} = k_{in}^1 \times \left(1 + \frac{E_{max}^1 C_e^1}{EC_{50} + C_e^1}\right) - k_{out}^1 \times E_1 \\
\frac{dE_2}{dt} = k_{in}^2 \times \left(1 + \frac{E_{max}^2 C_e^2}{EC_{50} + C_e^2}\right) - k_{out}^2 \times E \\
E = \alpha E_1 + \beta E_2
\end{cases}
$$

where C_e^i corresponds to the concentration of the ith component within the effect compartment, C_1^i denotes the concentration of the ith component in the central compartment, and k_e^i is the rate constant of the ith component; and k_{in}^i corresponds to the input rate constant for the ith component, k_{out}^i indicates the output or degradation rate constant for the ith component, E_{max} is the maximum effect of the ith component, and EC_{50} is the ith component concentration that yields 50% of the maximum effect size.

File S3: Hierarchical Optimization Algorithm

For the coupled PK stage, data from co-administration experiments are used to identify parameters that capture the mutual influence of each component. The objective function for this estimation process is designed to minimize the weighted residual sum of squares for each agent, defined as

$$min\,AIC = n_{pk} \sum_{j=1}^{2} ln\left(R_{ej}\right) + 2P_{pk} \ ,$$

where n_{pk} denotes the number of experimental data points, R_{ej} corresponds to the

weighted residual sum of squares for the jth component, and P_{pk} is the number of PK model parameters.

Model state and parameter estimation results need to follow basic physiological and pharmacokinetic rules. Hierarchical optimization algorithms follow this standard in the optimization process. All elimination and intercompartmental transfer rate constants of the coupled PK model take non-negative values. These values keep drug elimination direction and substance transport between central and peripheral compartments consistent with pharmacokinetic definitions. Numerical limits are set for parameters and model structures. Simulation results show non-negative drug concentration values in all compartments. The central and peripheral compartments are constrained to maintain pharmacokinetically consistent intercompartmental transfer and non-negative drug concentrations. Single-component PK data and actual drug concentration ranges after combined medication define the valid scope of coupled parameters. Special parameter combinations will produce low objective function values. These combinations do not match real physiological conditions and get excluded. All parameters need to meet the following constraint standards as

$$\begin{cases} 0 < k_{10} < 1 \\ 0 < k_{12} < 1 \\ 0 < k_{21} < 1 \end{cases} \quad \text{and} \quad \begin{cases} \alpha_{10} > \frac{-k_{10}^2}{max(C_1^1)}, \alpha_{12} > \frac{-k_{12}^2}{max(C_2^1)}, \alpha_{21} > \frac{-k_{21}^2}{max(C_2^1)} \\ \beta_{10} > \frac{-k_{10}^1}{max(C_1^2)}, \beta_{12} > \frac{-k_{12}^1}{max(C_1^2)}, \beta_{21} > \frac{-k_{21}^1}{max(C_2^2)} \end{cases}$$

For the coupled PD model, the objective function is defined as

$$\min AIC = n_{pd} ln(R_{pd}) + 2P_{pd}$$

where n_{pd} denotes the number of pharmacodynamic observations, P_{pd} corresponds

to the number of model parameters, and R_{pd} is indicative of the weighted residual sum of squares for pharmacodynamics.

For the PK-PD model, the effect-compartment rate constant k_e^i, the input rate constant k_{in} and the output rate constant k_{out} of the indirect response model are all constrained to positive values, so as to ensure that the dynamic equilibration of the effect-compartment concentration with the central-compartment concentration, as well as the processes of drug-effect onset and dissipation, proceed in an appropriate temporal direction. The parameters E_{max}^i, EC_{50}^i and those related to the combined effect are also confined within pre-specified pharmacologically reasonable ranges, in order to avoid negative effects, aberrant amplification, or other model behaviors that lack biological interpretability. Therefore, these parameters are required to satisfy the following constraints as

$$0 \leq k_e^i, k_{in}, k_{out} \leq 1 \quad i = 1,2.$$

$$\begin{cases} E_0^i > 0 & i = 1,2 \\ E_{max}^i > 0 & i = 1,2 \\ \lambda_i > 0 & i = 1,2 \\ EC_{50} > 0 & i = 1,2 \end{cases}$$

The PSO parameters were fixed at $\omega = 0.5$, $c_1 = 1.5$, and $c_2 = 1.4$, with a maximum of 200 iterations, and these settings were kept unchanged throughout all model estimations. These settings were selected based on preliminary convergence testing. A systematic sensitivity analysis of the PSO hyperparameters themselves was not performed. Instead, the numerical stability and robustness of the optimization procedure were further evaluated using 100 repeated optimizations with different random initial conditions. The corresponding results are presented in Section 3.4.

File S4: Comparison between Conventional and Coupled PK–PD Models

This study checked shifts in model fitting data with the coupled structure added. It made quantitative comparisons between traditional non-coupled models and newly designed coupled models. Identical experimental data were adopted for estimation of both model types. The same observed variables and error structures were applied for estimation of both model types. For calculation of AICc and BIC, n was defined as the total number of available animal-level observations included in the corresponding model comparison (n=144 for the joint PK model and n=72 for each PK-PD endpoint). Three metrics served as the model evaluation standard. The metrics include the corrected Akaike Information Criterion (AICc), Bayesian Information Criterion (BIC), and Likelihood Ratio Test (LRT). The AICc metric achieves a balance between model fitting quality and parameter quantity. The BIC metric delivers stricter restrictions on complex model structures. The LRT method works for significance verification. It verifies fitting improvements from newly added coupling parameters in nested model settings. The coupled PK model adds six concentration-dependent coupling parameters. These parameters are updated from the traditional PK structural framework. The coupled PK–PD model sets two extra coupling parameters and. These parameters are added on the original PD model foundation.

The specific model comparison results are presented in Table A1. For the PK model, the log-likelihood values of the conventional model and the coupled model were −128.40 and −126.20, respectively. This indicates that the likelihood improved after

introducing the coupling terms. However, because the coupled PK model added six free parameters, its AICc increased from 273.87 to 283.66, with a $\Delta AICc$ of 9.79. The BIC also increased from 296.56 to 321.98. The LRT results showed that the improvement in fit after adding the six coupling parameters did not reach statistical significance ($\chi^2 = 4.40,\ df = 6,\ P = 0.623$). These findings suggest that, under the current data conditions, the gain in fitting information from the coupled PK structure was insufficient to compensate for the increased model complexity due to the additional parameters. Therefore, from the perspectives of pure statistical model selection and parsimony, the conventional PK model exhibited a more distinct advantage.

For the PK-PD model, the differences between the two types of models were relatively small. For Caspase-3, the AICc values of the conventional PK-PD model and the coupled PK-PD model were 176.11 and 177.35, respectively, with a $\Delta AICc$ of 1.24. For HIF-1α, the corresponding AICc values were 168.71 and 169.55, with a ΔAICc of 0.84. The $\Delta AICc$ values for both comparisons were less than 2, indicating that the conventional model and the coupled model received similar levels of data support under the AICc criterion, with no clear superiority of one model over the other. Meanwhile, the LRT results for both Caspase-3 and HIF-1α did not reach statistical significance (Caspase-3: $\chi^2 = 3.60,\ df = 2,\ P = 0.165$; HIF-1α: $\chi^2 = 4.00,\ df = 2,\ P = 0.135$). The BIC favored the conventional model with fewer parameters in both PD endpoints, further reflecting the impact of complexity penalty on model selection outcomes.

These results do not support interpreting the coupled model as a replacement for the conventional PK-PD model on the basis of superior statistical fitting performance. At the PK level, the information criteria indicate that the introduction of six additional coupling parameters incurs a relatively substantial complexity penalty. At the PD level, the two model structures receive generally comparable support from the data, while the additional coupling parameters likewise do not produce a statistically significant improvement in model fit. Accordingly, the coupled model is positioned in this study as a mechanistic extension of the conventional PK–PD framework. Its primary value lies not simply in reducing information criteria or improving goodness of fit, but in explicitly quantifying potential PK and PD interactions between HSYA and CA while retaining an adequate description of the experimental data, thereby providing information on inter-component interactions that cannot be directly obtained from a conventional non-coupled model.

Table S1. Comparison between conventional and coupled PK/PK–PD models

Model component	Model	k	logL	AIC	AICc	BIC	ΔAICc	LRT	df	P
PK	Conventional model	8	-128.40	272.80	273.87	296.56	0.00	—	—	—
PK	Coupled model	14	-126.20	280.40	283.66	321.98	9.79	4.40	6.00	0.623
Caspase-3	Conventional PK–PD model	5	-82.60	175.20	176.11	186.58	0.00	—	—	—
Caspase-3	Coupled PK–PD model	7	-80.80	175.60	177.35	191.54	1.24	3.60	2.00	0.165
HIF-1α	Conventional PK–PD model	5	-78.90	167.80	168.71	179.18	0.00	—	—	—
HIF-1α	Coupled PK–PD model	7	-76.90	167.80	169.55	183.74	0.84	4.00	2.00	0.135
